# Wireless, miniaturized, semi-implantable electrocorticography microsystem validated in vivo

**DOI:** 10.1038/s41598-020-77953-8

**Published:** 2020-12-04

**Authors:** Keivan Keramatzadeh, Ali Kiakojouri, Mohammad Sadegh Nahvi, Yousef Khazaei, Ali Feizi-nejad, Mohammad Hossein Maghami, Reza Mohammadi, Mohammadali Sharifshazileh, Soraya Nasiri, Farhad Akbari Boroumand, Ebrahim Nadimi, Mahmoud Rezaei, Amir Shojaei, Javad Mirnajafi-Zadeh, Amir M. Sodagar

**Affiliations:** 1 Research Labarotory for Integrated Circuits and Systems (ICAS), Faculty of EE, K.N. Toosi University of Technology, Tehran, Iran; 2 Faculty of Electrical Engineering, Shahid Rajaee Teacher Training University, Tehran, Iran; 3 Department of ECE, University of Waterloo, Waterloo, ON Canada; 4 Institute of Neuroinformatics, University of Zurich and ETH Zurich, Zürich, Switzerland; 5 Faculty of EE, K.N. Toosi University of Technology, Tehran, Iran; 6 Department of Physiology, Faculty of Medical Sciences, Tarbiat Modares University, Tehran, Iran; 7 Department of EECS, York University, Toronto, ON Canada

**Keywords:** Biomedical engineering, Electrical and electronic engineering, Cognitive neuroscience

## Abstract

This paper reports on the design, development, and test of a multi-channel wireless micro-electrocorticography (µECoG) system. The system consists of a semi-implantable, ultra-compact recording unit and an external unit, interfaced through a 2.4 GHz radio frequency data telemetry link with 2 Mbps (partially used) data transfer rate. Encased in a 3D-printed 2.9 cm × 2.9 cm × 2.5 cm cubic package, the semi-implantable recording unit consists of a microelectrode array, a vertically-stacked PCB platform containing off-the-shelf components, and commercially-available small-size 3.7-V, 50 mAh lithium-ion batteries. Two versions of microelectrode array were developed for the recording unit: a rigid 4 × 2 microelectrode array, and a flexible 12 × 6 microelectrode array, 36 of which routed to bonding pads for actual recording. The external unit comprises a transceiver board, a data acquisition board, and a host computer, on which reconstruction of the received signals is performed. After development, assembly, and integration, the system was tested and validated in vivo on anesthetized rats. The system successfully recorded both spontaneous and evoked activities from the brain of the subject.

## Introduction

Extra-cellular recording of brain activities is of increasing interest in a variety of applications, namely advanced neuroscience research, diagnosis and treatment of neural diseases and disorders, and neuro-prosthetic applications^1^. There are three approaches that are commonly used for extracellular electrical recording of neuronal activities: *electroencephalography* (*EEG*), *electrocorticography* (*ECoG*), and *intra-cortical recording* (*ICR*). The EEG approach is nowadays widely used for clinical purposes as well as in a wide variety of brain-machine interfacing (BMI) application areas, from brain-operated cognitive rehabilitation and computer games^2^ to brain-controlled autonomous vehicles^3^. Despite its rather low resolution of recording, this approach is well embraced in applications of public interest as it is non-invasive. The systems developed for intra-cortical neural recording, also known as brain implants, have attracted tremendous interest in cutting-edge neuroscience research, prosthetic devices, and therapeutic applications because of the fine recording resolution (both temporal and spatial) they provide. As some of the major works that paved the road towards functionally-mature intra-cortical neural recording brain implants one can point to the first self-sufficient, 32-channel, microsystem reported in^4^, the 64-channel fully-wireless integrated microsystem presented in^5^, and the hybrid (integrated circuits + PCB) 100-channel systems introduced in^6,7^. The ECoG approach is the recording of neuronal activities from the surface of the brain, which has recently attracted significant attention in neural interfacing. This is mainly because of the finer spatial and temporal resolution and higher signal-to-noise ratio this approach provides compared to EEG, and also being less invasive compared to intra-cortical neural recording^8^. In general, electrocorticography offers advantages such as sufficiently-good signal quality, rather easy surgery and implantation, and efficient interfacing to the external world, which makes it a proper candidate for a wide variety of application areas, including brain-machine interfacing^9^, neural prostheses^10,11^, epileptic focus localization^12^, and brain mapping^13^.

From the perspective of physical development, electrocorticography systems have been developed with three different approaches: In^14,15^, an electrode array is connected to a rack-mount/benchtop commercially-available neural recording lab equipment, resulting in a *distributed* recording unit. In^16–18^, a *compact/ultra-compact* recording device is developed using an electrode array and a small (palm-size or smaller) electronic unit prototyped using off-the-shelf electronic and electrical components. Such devices can be used as wearable and in some cases semi-implantable recording tools. The works reported in^19,20^ are examples of *integrated ECoG microsystems*, realized as a result of the integration of all parts (or at least the electronic circuitry) of the recording unit on a single chip. From the standpoint of the size and coverage of electrode array, there are two major types of ECoG systems: (*i*) *Macro-ECoG systems* with rather large electrode size (mm-cm) and wide coverage (covering rather large areas of the brain) ^21^, and (*ii*) *Micro-ECoG* (*μECoG*) *systems*, with sub-mm electrodes allowing for cortical recording of neuronal activities with high spatial resolution ^22^.

**Figure 1:**
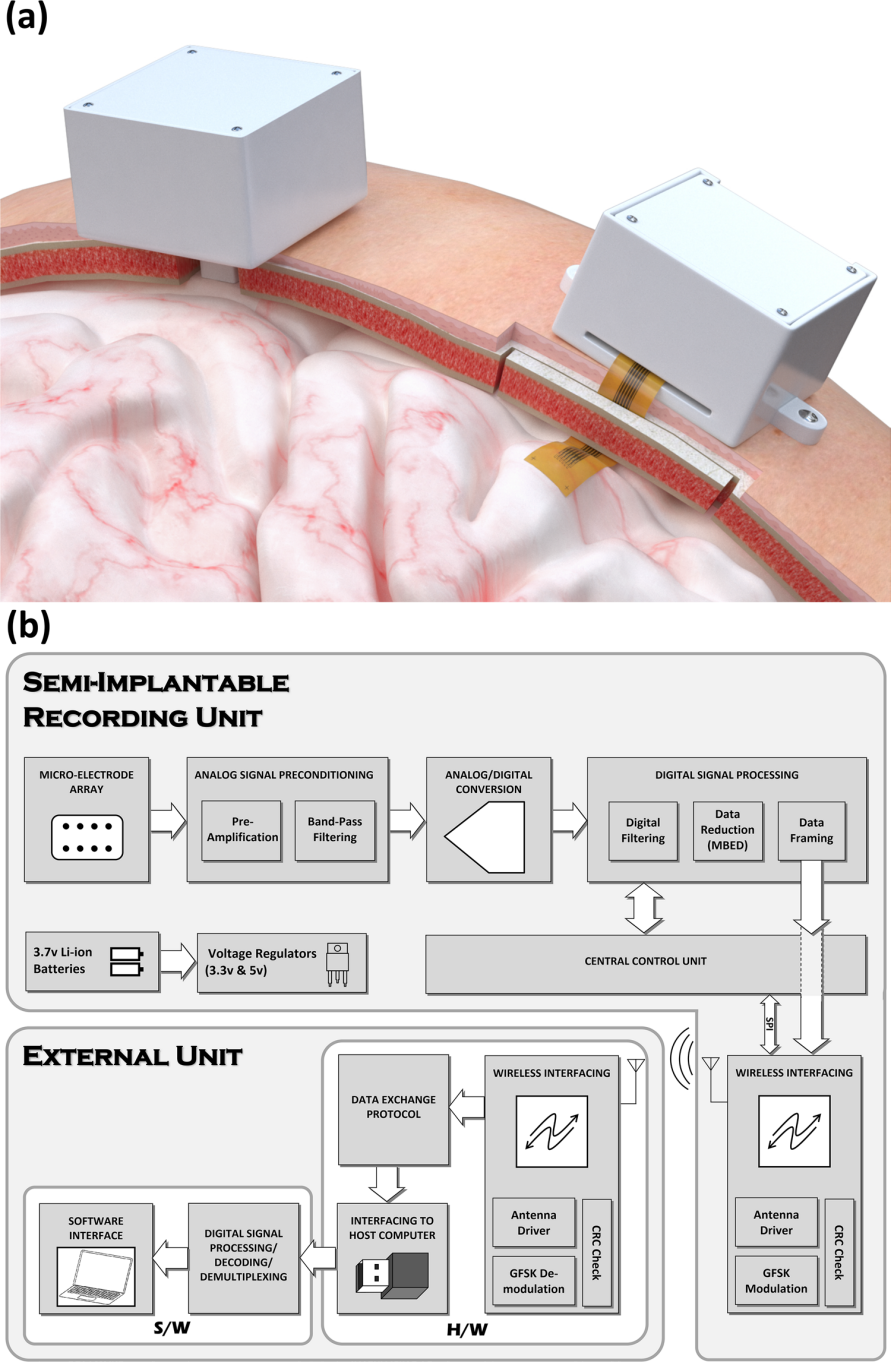
(**a**) Illustration of two versions of the ultra-compact minimally-invasive ECoG recording microsystem and their placement for sub-dural recording. (**b**) Functional block diagram of the system, comprising the recording unit and the external unit interfaced through wireless connection.

This paper reports on the design and development of two versions of a semi-implantable ultra-compact μECoG microsystem, and validation of their operation in vivo. This microsystem (*recording unit*) exchanges data with an external host (*external unit*) through radio-frequency wireless communication. Figure 1a illustrates both versions of the recording unit as well as their placement for sub-dural ECoG recording. *Version-1 of the recording unit* uses a rigid microelectrode array (MEA), which is connected at the bottom of the cubic package. The MEA in this version comes in touch with the surface of the brain through a burr hole made in the skull of the subject. The MEA in *Version-2* is fabricated on an organic flexible substrate connected to the electronics through a flexible ribbon cable. In this version, the flexible MEA structure sticks out of the recording unit through a slot on a side of the cubic package. To implant the flexible MEA, a small piece of the skull needs to be removed, the MEA is placed on the surface of the brain, and the skull piece is put back in order to close the exposed part of the brain.

## Materials

### Manufacturing of the ultra-compact semi-implantable microsystem

The semi-implantable ultra-compact recording unit consists of electronic circuitry assembled on a stack of four 2.5 cm × 2.5 cm and one 1.1 cm × 1.8 cm printed circuit boards (PCBs), two small-size 3.7-V, 50 mAh lithium-ion batteries, two versions of microelectrode array, and an RF-transparent bio-compatible package. Total weight of Version-1 of the system (the heavier version) including the package, microelectrode array, all electronic circuits, and the batteries is 27 g. As shown in the simplified block diagram of Fig. 1b, electrocorticogram signals are concurrently recorded using a microelectrode array followed by a signal preconditioning block. This block prepares the signals for digitization by pre-amplification and DC-level shifting using per-channel, low-power, low-noise amplifiers with input refered noise of $8 {\text{nV}}/\!\sqrt {{\text{Hz}}} @ 1 {\text{kHz }}$ and programmable gain of 1–10,000 (Analog Devices AD8222). The preconditioned signals are then sampled at a rate of 1 ksps and digitized with a resolution of 10 bits (Texas Instrument analog-to-digital converter ADS1298). The digitized neural signals along with error control bits are then packaged in the form of serial data frames, and transmitted off the recording unit through wireless connection. For this purpose, the NORDIC nRF24L01 radio frequency transceiver module with a carrier frequency of 2.4 GHz, a maximum bit rate of 2 Mbps, and maximum transmission range of 50 m is used. Operation of the entire recording unit is administered using a microcontroller, running at a clock frequency of 16 MHz (ATMEL ATmega32). According to experimental results, operated using a pair of batteries, the semi-implantable recording unit is capable of continuous operation for about 1 h.

#### Rigid MEA

**Figure 2:**
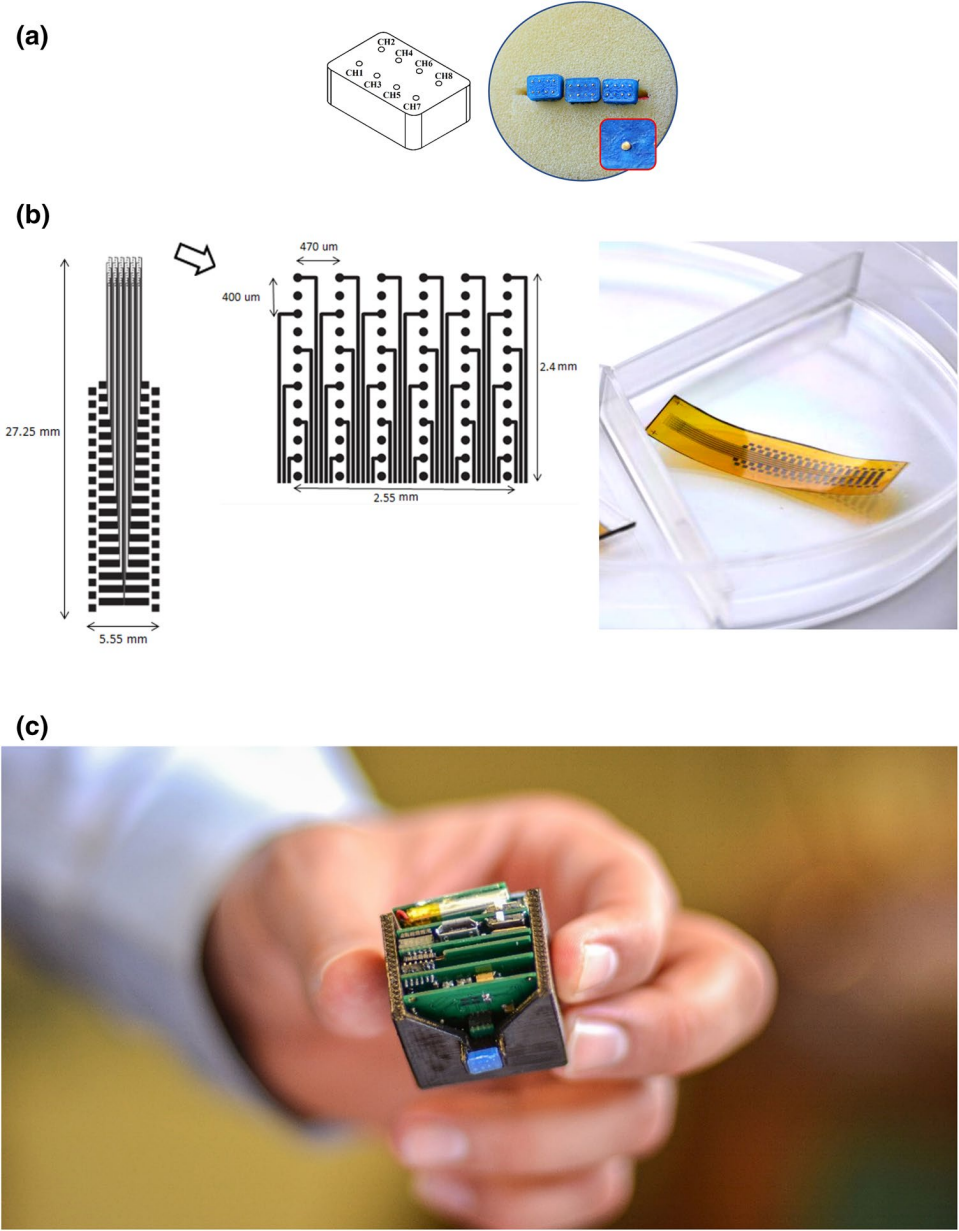
Two versions of the microelectrode array used in this work. (**a**) The rigid microelectrode array implemented on a PLA substrate with gold electrodes. (**b**) The flexible microelectrode array microfabricated on a polyimide substrate with titanium electrodes. (**c**) Photograph of Version-1 of the actual implantable unit after assembly and integration.

The MEA developed for Version-1 of the recording unit is a 2 × 4 array of gold electrodes fabricated on a rectangular-shape rigid substrate. The substrate is made of Polylactic Acid (PLA), which is a biocompatible material^23^, and measures 6 mm (L) × 4 mm (W) × 2 mm (H). In this version, in addition to being in close proximity of/in touch with the surface of the brain from the front side, the electrodes are electrically accessible from the back side of the substrate. Electrode configuration, and photograph of the finished electrode array are shown in Fig. 2a.

#### Flexible MEA

Second version of the MEA is a 12 × 6 array of titanium electrodes microfabricated on the surface of a flexible polyimide substrate, half of which are electrically accessible through bonding pads. Electrodes are of circular shape with a diameter of 100 μm. Layout of the electrode array and photo of the electrode array after the completion of the microfabrication process are shown in Fig. 2b. As shown in the layout, vertical and horizontal pitch size of the electrodes (the ones that are actually connected) is 400 μm and 470 μm, respectively, and uniformly cover a rectangular area of 2.55 mm × 2.4 mm. Details of fabrication and characterization of the flexible MEA was published in^24^.

#### Packaged semi-implantable recording unit

Photograph of the packaged semi-implantable recording unit after assembly and integration inside the first version of the package is shown in Fig. 2c. It is worth noting that this package is intentionally fabricated in two separate pieces. As seen in the picture, one side of the package is removed in order for the easier placement of the electronic circuit boards in the package and more importantly to make it possible to connect the microelectrode array to the backside of the bottom-most board.

### External unit

The external unit comprises the receiver side of the RF link, a laptop host computer equipped with a data acquisition board, and a special-purpose software specifically developed for this system. On the data acquisition board, a microcontroller takes the digital data packets received from the RF link, and delivers them through a universal serial bus (USB) port to the host computer in the form of a serial bit stream with universal asynchronous receive/transmit (UART) protocol. Decoding, de-multiplexing, and processing of the digital data are all performed on the host computer. This software is capable of real-time streaming/plotting the signals being recorded as well as locally storing them for further studies and offline processing.

## Experiments and results

### Preliminary tests, validation, and characterization

**Figure 3:**
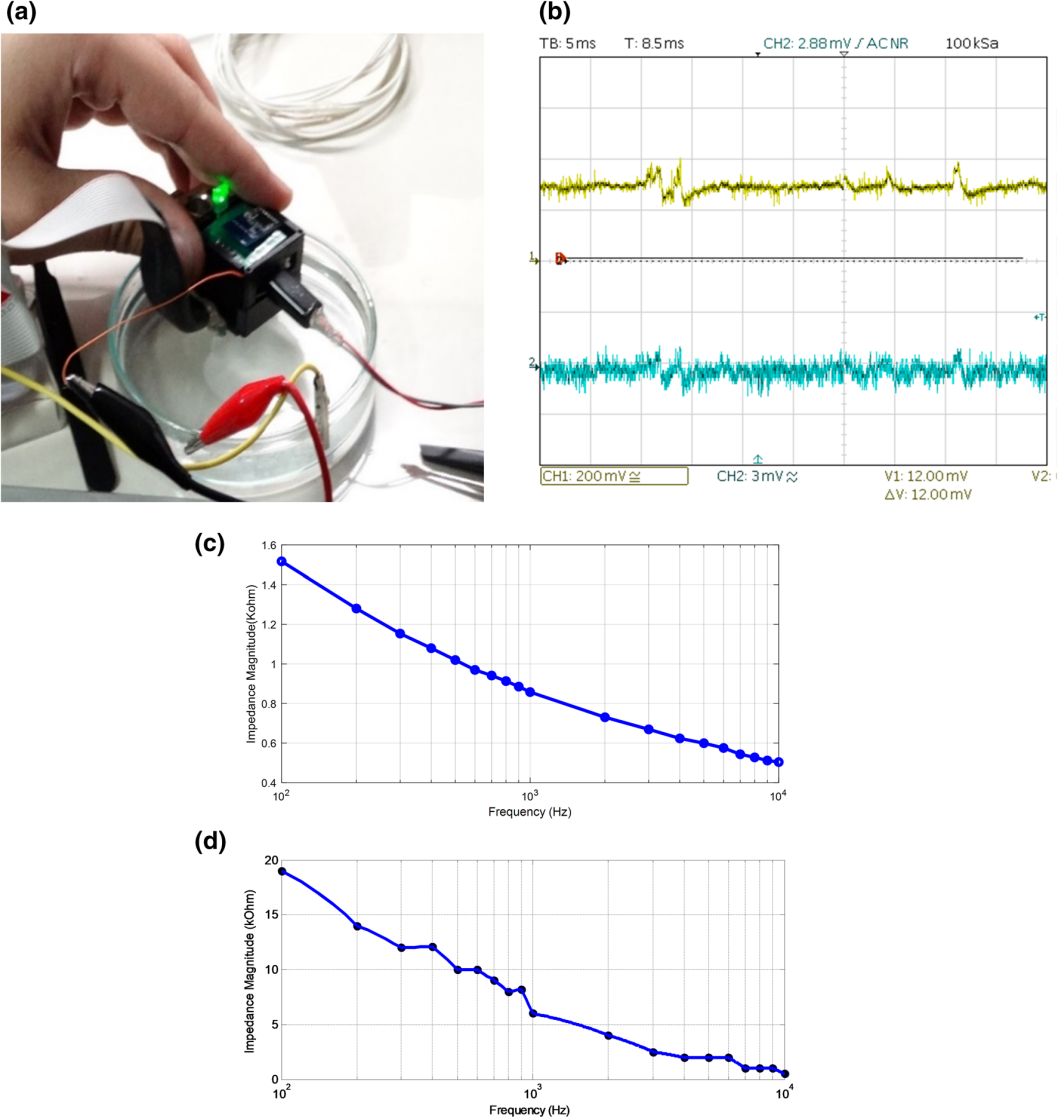
(**a**) Test and validation of the Version-1 recording unit in saline, (**b**) oscilloscope screenshot for the test shown in (**a**) with the input prerecorded ECoG signal at the bottom and the output amplified signal at the top, (**c**) impedance spectroscopy for the rigid microelectrode array, (**d**) impedance spectroscopy for the flexible microelectrode array.

To verify and assess its operation, first, different parts of the system, and then the complete integrated system were tested, validated, and characterized. Sample pictures for such tests are presented in Fig. 3. As shown in Fig. 3a for the Version-1 recording unit, the electrode array was immersed in a dish of saline, to which a pre-recorded electrocorticogram signal was applied as the input. Figure 3b shows the input signal (bottom) and the amplified and low-pass filtered signal at the output (top). In-vitro impedance spectroscopies for both rigid and flexible electrode arrays in saline medium are shown in Fig. 3c,d. According to the experimenal results, impedance of the rigid and microfabricated flexible electrodes are ~ 0.9 kΩ and ~ 6 kΩ at the frequency of 1 kHz, respectively.

### In-vivo experiments

#### Preparation of the subject

**Figure 4:**
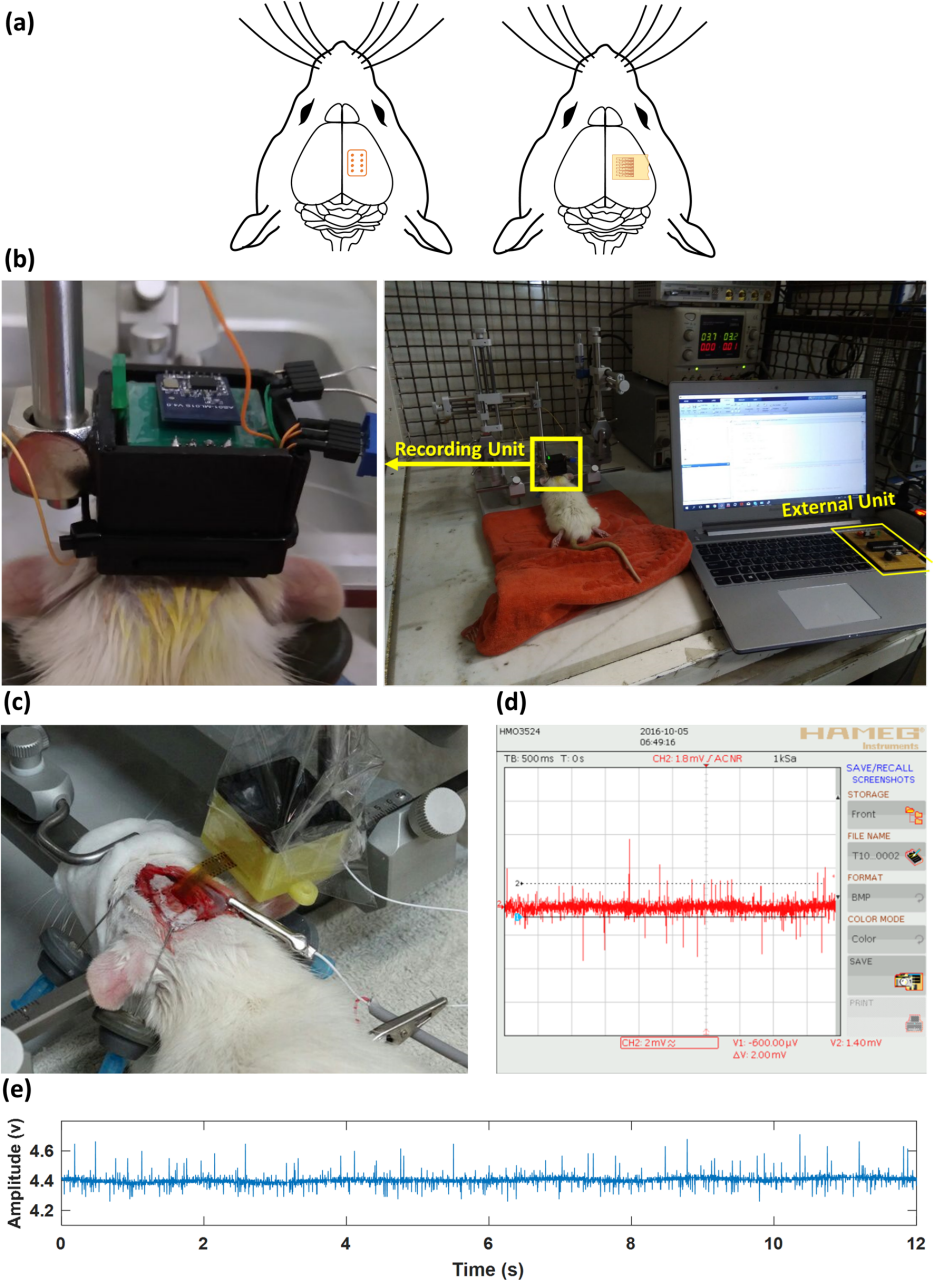
(**a**) Placement of both versions of the microelectrode array on the somatosensory cortex of the brain of the subject. (**b**) In-vivo experimental setup inside a Faraday cage for ECoG recording from the brain of an anesthetized rat. (**c**) Test and validation of the flexible microelectrode array in vivo*.* (**d**) A sample ECoG signal recorded in vivo on one electrode of the flexible microelectrode array. (**e**) A sample ECoG signal recorded in vivo on one electrode of the rigid microelectrode array.

Acute animal experiments were conducted in vivo at the Epilepsy Laboratory at the Faculty of Medical Sciences, Tarbiat Modares University, Tehran, Iran. For this purpose, adult male Wistar rats (3 months old) that were obtained from Pasteur Institute of Iran were maintained in a colony room kept at 23 ± 2 °C temperature on 12:12 light:dark schedule (lights on at 7:00 A.M.) and permitted free access to food and water. All experiments were performed in accordance with the ethical guidelines set by “Research Ethics Committee of Faculty of Medical Sciences, Tarbiat Modares University that were completely coinciding with the “NIH Guide for the Care and Use of Laboratory Animals”^25^. The animal was anesthetized using a mixture of ketamine 10% (Alfasan, the Netherlands) and xylazine 2% (Alfasan, the Netherlands) (80 and 12 mg/kg, respectively; i.p. injection). In this situation, the anesthesia maintained for about 1 h. Then, the animal’s head was fixed in a stereotaxic frame (Stoelting, USA) while the body temperature was controlled by a rectal probe and a blanket under the rat. The head skin shaved and an incision was made in its midline to expose the skull. An area of about 5 mm × 7 mm of the skull above the animal’s somatosensory cortex in the right parietal lobe was removed by using micro-drill (Saeshin, Strong 204, Korea). The position of this area was determined according to the atlas of rat’s brain (Paxinos & Watson) at the coordination of 0.6–7.6 mm posterior to bregma and, 1–6 mm from the midline. Placement of the two versions of the MEA and the in-vivo experimental setup are shown in Fig. 4a. In this setup, the system and the anesthetized animal were placed inside a Faraday cage for electromagnetic noise shielding as shown in Fig. 4b.

#### Validation of microelectrode arrays

The rigid and flexible microelectrode arrays were both tested in vivo. Figure 4c shows pictures of the flexible MEA in the associated package, when it is used to record electrocorticogram signals from the brain of the subject in vivo. For these tests, electrode arrays were connected to commercial neural recording lab equipment. Sample signals recorded using the flexible and rigid MEA are shown in Fig. 4d,e.

#### Validation of the system

**Figure 5:**
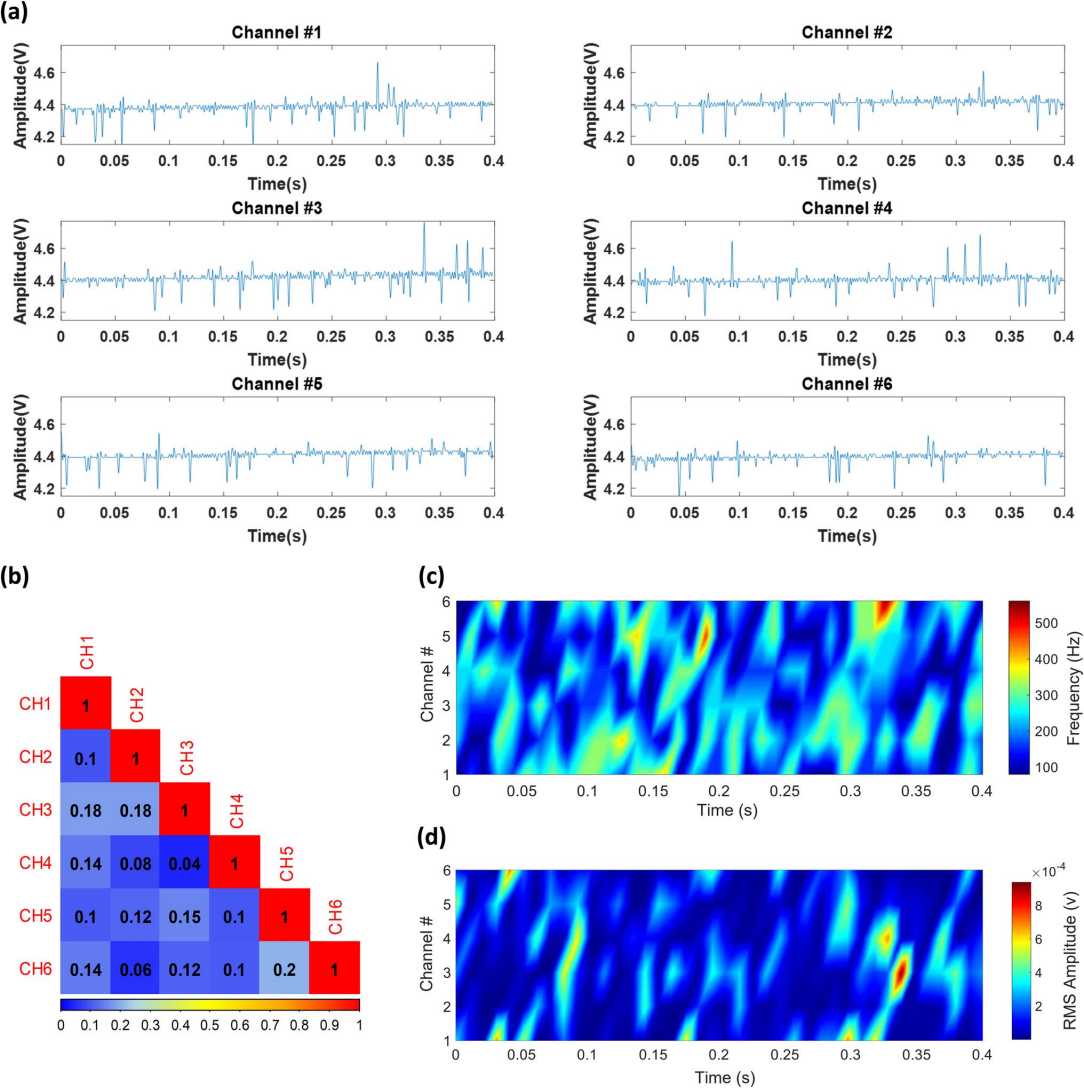
In-vivo recording of spontaneous neuronal activities from somatosensory cortex of the brain of an anesthetized rat. (**a**) Recorded signals in the time domain. (**b**) Correlogram of the recorded activities. (**c**) Spectrogram of the signals on all the 6 channels. (**d**) Colormap diagram of the RMS amplitude of the recorded signals.

**Figure 6:**
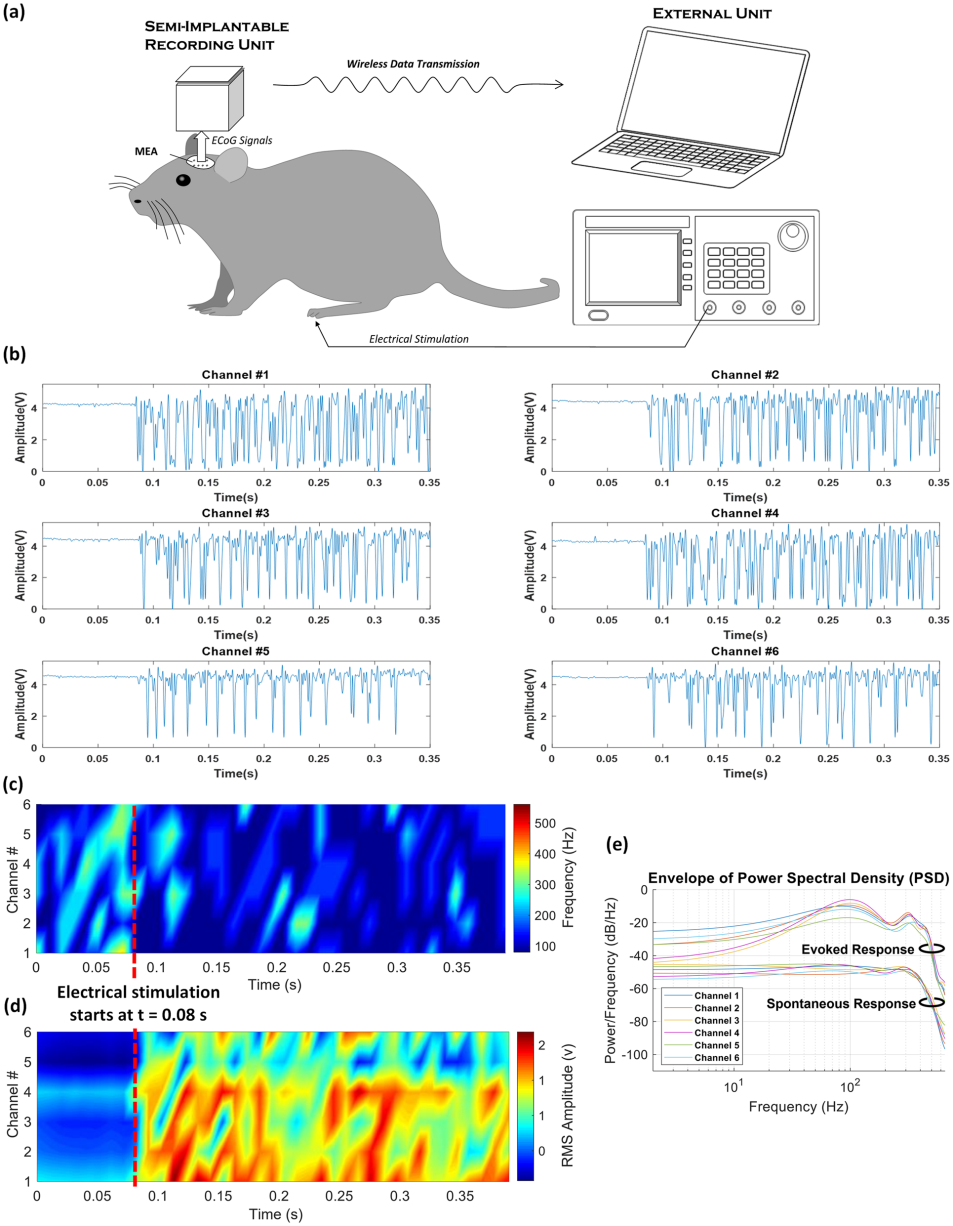
In-vivo recording of evoked activities. (**a**) Applying electrical stimulus to the hind paw of the subject, (**b**) recorded signals, (**c**) spectrogram, (**d**) colormap diagram of the RMS amplitude of the recorded signals, and (**e**) power spectral densities of the signals recorded on all the six channels in both spontaneous and evoked experiments.

(a) *Spontaneous activities*: As shown in Fig. 4b, the complete system with the rigid MEA was used for in-vivo recording from the brain of the subject. In this test, “spontaneous” activities were recorded from the contralateral somatosensory cortex of the subject with a gain of 1000 in 10-s sessions. A small slice of the recorded signal is presented in Fig. 5a. Signals on channels 7&8 were not presented due to their poor quality. The reason for the poor signal quality on these channels was that the associated electrodes did not come to close proximity of/in touch with the cortex. Small correlations between the signals recorded, as presented in the correlogram of Fig. 5b, is an indication of the existance of no considerable redundancy in the signals being recorded. Figure 5c shows the spectrogram of all the 6 channels, in which frequency content of the recorded spontaneous activities are spread up to around 300 Hz, as reported in the literature^16,17,26,27^. Colormap diagram for the RMS amplitude of all the 6 channels is shown in Fig. 5d. (b) *Evoked activities*: Next, as illustrated in Fig. 6a, the system was used to record “evoked” activities from the subject in response to applying electrical stimulation (50-μA rectangular pulses) to the hind paw of the animal to stimulate the somatosensory nerves. Figure 6b,c show the signals recorded in this experiment as well as their spectrogram, in which electrical stimulation starts at around t = 0.08 s. Colormap diagram for the RMS amplitude of the recorded activities is also shown in Fig. 6d. Power spectral densities of the signals recorded on all the six channels in both spontaneous and evoked experiments are shown in Fig. 6e. According to^28^, the rise in the evoked response spread from ~ 60 Hz up to ~ 200 Hz peaking at 100 Hz represents high-gamma activities.

## Conclusions and discussion

Two versions of an ultra-compact, minimally-invasive, semi-implantable µECoG system are presented. First version is an 8-channel system with a rigid microelectrode array. In this version of the system, the electrode array comes in touch with the cortex (or the dura layer) through a burr hole in the skull. Second version of the system uses a flexible microelectrode array with 72 recording sites, 36 of which are electrically accessible through bonding pads. Surgical approach to implant the flexible microelectrode array is to take a small piece of the skull away, place the microelectrode array on the surface of the brain (or on the dura layer for epi-dural recording), and put the removed piece of the skull back in order to close the opening. Both microelectrode arrays were validated in vivo. The recording unit was then integrated and packaged as a stand-alone device, and tested in vivo. Comprehensive tests of the electronic part of the implant was performed in the version of the system that contains the rigid microelectrode array. In-vivo experiments of the whole system included the recording of both spontaneous and evoked activities from the somatosensory cortex of anesthetized rats. Table 1 summarizes functional, physical, and electrical specifications of the system along with those for several other recent works for comparison.

**Table 1:** Summary of specifications for the system reported in this particle in comparison with those of other recent works.

		Sampling frequency (kHz)	No. of channels	Resolution (bit)	Power consumption	Data communication	Wireless data rate	Device size
Distributed ECoG systems	[15]	25	64	Up to 16	2.8 W*	Hardwired	-	15.5 cm × 9.9 cm (Eval. board size)*
	[14]	1	64	Up to 16	2.8 W*	Hardwired	-	> 15.5 cm × 9.9 cm (Eval. board size)*
Compact ECoG systems	[18]	1	128	10	140 mW	RF (Microsemi ZL70102)	515 Kbps	4.6 × 3.2 × 0.4 cm^3^
	[16]	1	64	12	75 mW	MICS band 402–405 MHz	450 Kbps	5 cm diameter antenna: 10cm^2^
Integrated ECoG systems	[19]	1	64	15	225 μW	RF link	1 Mbps	6.5 mm diameter
	[20]	1-31.25	16	8	365 μW	ISCOM	10 Kbps	1.3 cm^3^ volume
Ultra-compact ECoG systems	[17]	5	16/32	Up to 16	N/A	Hardwired	-	> 10 × 6 × 3 cm^3^
	This work	1	8/36/72	10	209 mW	RF link	2 Mbps	2.9 × 2.9 × 2.5 cm^3^

*These systems use Intan RHD2000 evaluation board for recording. Power consumption and device size are according to the catalog of this board.

According to the categorization presented earlier, the system reported in this article is an ultra-compact ECoG system. Compared with the state-of-the-art ultra-compact ECoG device recently reported in^17^, both systems are of almost the same order of recording channel count. Main advantages of our system, however, are as follows: (*i*) Our system is equipped with a high-speed RF telemetry link on one hand, and operated using high energy density, small-size batteries on the other hand, our recording unit is a stand-alone, fully-wireless device. This is while the work in^17^ is connected to the external setup through hardwired connection in order to receive electric power and exchange data. (*ii*) From the standpoint of physical size, our recording unit is more than 8 times smaller (21 cm^3^ vs. 180 cm^3^). (*iii*) Physical structure of our device (including the form factor of the device and the way the microelectrode arrays are integrated with the rest of the system) allows for the recording unit to be mounted on the head of the subject for freely-moving experiments.

## Methods

### Fabrication of rigid microelectrode array

**Figure 7:**
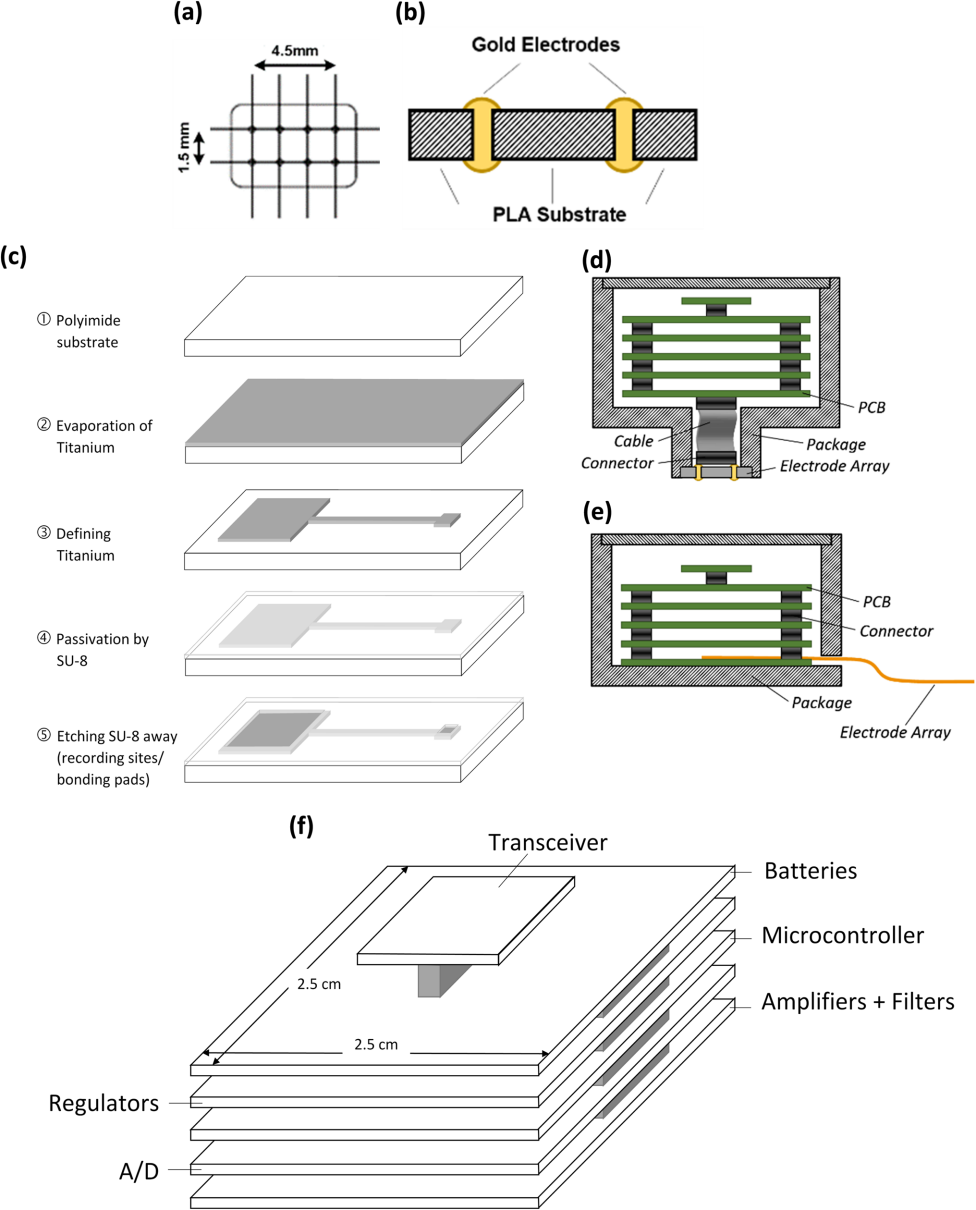
Development and integration of the semi-implantable recording unit. (**a**) The 3D-printed PLA substrate for the rigid MEA. (**b**) Cross-sectional view of the gold electrodes added to the rigid substrate. (**c**) Microfabrication process flow for the flexible MEA. (**d,e**) Illustration of the integration of Version-1 and Version-2 of the recording units, respectively. (**f**) The stacked PCB platform containing the electronic circuitry and batteries.

The rigid microelectrode array is fabricated in three steps: First, a PLA substrate with precisely-spaced hole marks is made by 3-dimensional (3D) printing. Then, as sketched in Fig. 7a, an array of 2 × 4 holes with a pitch size of 1.5 mm are made in the substrate by micro-drilling with a diameter of 350 µm. Subsequently, gold wires with a thickness of 300 µm are threaded through the holes, and finished on both sides of the substrate with the shape of round head rivets, as illustrated in Fig. 7b.

### Fabrication of flexible microelectrode array

Microfabrication process flow for the flexible microelectrode array is shown in Fig. 7c. The process starts with a flexible polyimide substrate, with the thickness of 25 μm. A layer of titanium is deposited on the surface of the substrate by evaporation. This layer is then patterned in order to form electrodes (recording sites), bonding pads, and also interconnects between the electrodes and bonding pads. Surface of the structure is subsequently passivated by a layer of SU-8*,* which is a biocompatible^29^ and transparent material, and electrically performs as an insulator. Because of having a Young’s modulus close to that of polyimide (Y_SU-8_ = 4.02 GPa and Y_Polyimide_ = 1.3–4 GPa), the SU-8 coating does not reduce the flexibility of the microelectrode array^30,31^.

### Integration and packaging

To encase all parts of the semi-implantable recording unit, a cubic-shape package is designed in two versions. As shown in Fig. 7d,e, the first version integrates the electronic boards and batteries with the rigid electrode array, and the second version is designed to integrate the flexible array with other parts of the recording unit. Both packages are fabricated by 3D printing of PLA. First version of the package consists of two concentric cubes, measuring 2.9 cm (W) × 2.9 cm (L) × 2.5 cm (H) and 5 mm(W) × 7 mm (L) × 5 mm (H) with a total weight of 6 g. The smaller cube (facing down) embodies the rigid array in such a way that the electrodes can easily come in touch with the brain tissue. The electrode heads on the back side of the rigid substrate are connected to a small connector using silver paste. Through a flexible cable, the signals are taken from the microelectrode array to the electronics on a PCB platform. The larger cube (facing up) contains the PCB platform and batteries. Second version of the package is only one cubic container encasing the PCB platform and batteries with a slot on one of the sidewalls that lets the flexible microelectrode array stick out and be flexibly bent down in order to come in touch with the surface of the brain. Two screw holes are envisioned on the side of the package in order to mount and secure the package on the skull using two small orthopedic self-drilling screws. Both versions of the package come with caps that are screwed on the top side of the package.

#### The PCB platform

As shown in Fig. 7f, the PCB platform is a vertically-stacked PCB structure, integrating multiple electronic boards and the batteries in a compact way. The bottom-up arrangement of the boards is in the natural order the signals need to go through: Pre-amplification, analog-to-digital conversion, digital handling and processing of the signals using a microcontroller, and finally wireless transmission. There are also two boards holding the batteries and regulators.

### Digital signal processing

Electrocorticogram signals recorded on neighbouring channels usually share similar baseline variations, especially when they are recorded using microelectrode arrays^32^. Riding on this shared background, most of the recorded information appear in channel-specific signal variations. The co-varying background levels are usually of large amplitude and rather low frequency, while channel-specific activities are of much smaller amplitude and higher frequency content. In this work, elimination of the redundant baseline variations from the signals concurrently recorded on multiple neighboring channels (recently proposed by the authors in^33^) is the basis for some extent of data reduction without losing any information. This idea can also be employed for lossless spatial data redundancy reduction in the brain implants that are developed for intra-cortical neural recording (using microelectrode arrays)^34^. In this case, local field potentials are the co-varying common components that redundantly appear on multiple channels.

Referred to as the “*Multichannel-Baseline-Extraction Decomposition* (*MBED*)” hereafter, this method first extracts a *common baseline* signal:

1 $$X_{B.L.} \left( k \right) = \frac{1}{N}\mathop \sum \limits_{i = 1}^{N} X_{i} \left( k \right)$$

in which *N* is the number of channels, and *X*_*i*_(*k*) is the signal on the *i*-th channel. It is assumed that all the signals are digitized with a resolution of *M* bits (in the format of integer unsigned binary numbers). Then, channel-specific *difference components* are obtained by subtracting the common baseline signal from the original signals on all the channels:

2 $$\Delta X_{i} \left( k \right) = X_{i} \left( k \right) - X_{B.L.} \left( k \right) \left( {i = 1, 2, \ldots , N} \right).$$

Being of much smaller amplitude compared to the original signals, the difference components are represented by a smaller number of bits. The key point in the MBED method is to save considerable data transfer rate by expressing the neural signals using one common baseline component and per-channel difference components (rather than the original signals).

#### Data reduction

Assuming that the common baseline signal is of rather large amplitude variations in the time domain (in comparison to amplitude fluctuations of the channel-specific difference components), common baseline samples will, therefore, be of the same width as the original signal samples (i.e., *M* bits), and the difference component words will be shorter, say *m* bits. The data reduction achieved using the MBED technique is quantified by a *bit-rate reduction*, *BRR*, of:

3 $$BRR\% = \frac{{\left( {Total\, \# of\, bits \,before\, reduction} \right) - \left( {Total\, \# \,of \,bits \,after\, reduction} \right)}}{Total\, \# \,of\, bits \,before \,reduction} \times 100\% = \frac{{\left( {N \times M} \right) - \left( {M + \left( {N \times m} \right)} \right)}}{{\left( {N \times M} \right)}} \times 100\% .$$

Rewriting Eq. (3) as:

4 $$BRR\% = \left( {1 - \frac{1}{N} - \frac{m}{M}} \right) \times 100\% ,$$

the maximum achievable BRR is evidently a function of the ratio of the amplitude swing of the difference components to that of the common baseline signal (*m/M*), and of course the number of recording channels. As a practical example, for the multi-channel ECoG recordings reported in^35^, average *m/M* ratio is ~ 0.5. With *M* = 10 and *m* = 5, the MBED technique results in a BRR% of around 37.5%. It should be noted that this reduction is lossless and resulted merely from the removal of the spatial redundancy that exists in the signals being recorded. On top of this “spatial compression”, “temporal compression” techniques can be used in order to achieve further data reduction.

### In-vivo experiment protocol

All experiments were performed in accordance with the ethical guidelines set by “Research Ethics Committee of Faculty of Medical Sciences, Tarbiat Modares University” that were completely coinciding with the “NIH Guide for the Care and Use of Laboratory Animals”^25^. The experimental protocol was approved by the “Research Ethics Committee of Faculty of Medical Sciences, Tarbiat Modares University”.

## Data Availability

The datasets generated and analyzed during the current study are available from the corresponding author on reasonable request.
